# Women’s empowerment in agriculture and productivity change: The case of Bangladesh rice farms

**DOI:** 10.1371/journal.pone.0255589

**Published:** 2021-08-04

**Authors:** Mohammad Hasan Mobarok, Theodoros Skevas, Wyatt Thompson

**Affiliations:** Division of Applied Social Sciences, University of Missouri, Columbia, MO, United States of America; International Maize and Wheat Improvement Centre: Centro Internacional de Mejoramiento de Maiz y Trigo, MEXICO

## Abstract

Using productivity change as a measure of farm economic performance, we analyze the relationship between women’s empowerment in agriculture and farm productivity change and its components, which include efficiency change, technological change, and scale efficiency change. A non-parametric Malmquist approach is used to measure farm specific productivity change and its decomposition. We use a bootstrap regression to analyze factors that cause differences in productivity change and its components, testing, in particular, the role women’s empowerment plays. The empirical application focuses on a sample of Bangladesh rice farms over the crop cultivation period 2011 and 2014. Results suggest that improvements in women’s empowerment in agriculture were associated with higher levels of productivity change, efficiency change, and technical change, while they had no impact on scale efficiency change. We find that empowering women, specifically, improving their ability to make independent choices regarding agricultural production had a statistically significant positive association with productivity change, efficiency change, and technical change. We also find that lowering the gender parity gap is positively related with improving productivity of the sample farms.

## 1. Introduction

Agricultural productivity growth is an important component of any development strategy [1,2]. At the farm level, productivity growth can be viewed as an indicator of a farm’s ability to persist in an environment where market, regulatory, and environmental pressures may reduce its competitiveness and even lead to farm exit. Given the importance of agricultural productivity growth for development and agriculture’s economic sustainability, extensive research has been put into measuring productivity change and understanding the factors that drive it. Prominent among the factors that have been investigated to better understand changes in productivity are farm and farmer characteristics, farm policies, and climatic conditions [3–5]. Women’s empowerment and its relationship with farm-level productivity, have not been studied in depth so far, although they might be relevant, especially in developing countries where women make up for 43% of the agricultural labor force [6].

To date, a number of studies have looked at the effect of women’s empowerment in agriculture on farm performance measures, but not specifically on farm productivity change. For example, studies by Bozoğlu and Ceyhan [7] and Seymour [8] examined the effect of women’s empowerment on farm technical efficiency. Bozoğlu & Ceyhan [7] found that greater participation of women in decision-making and labor activities led to higher levels of farm technical efficiency. Although Bozoğlu and Ceyhan [7] do not explicitly use the term “women’s empowerment”, their measure of women’s participation in farm decision-making can be considered a measure of women’s empowerment. On the other hand, Seymour [8] found only a weak positive relationship between women’s empowerment (as measured using Women’s empowerment in Agriculture Index (WEAI) developed by Alkire et al. [9]) and technical efficiency. Besides technical efficiency studies, another stream of relevant literature has looked at the effect of women’s empowerment on crop output quantity. More specifically, Diiro et al. [10] employed an abbreviated version of WEAI to study the effect of women’s overall empowerment and its individual indicators on maize yield of farm households in western Kenya and found a positive effect on yield. Another study [11] in this area used WEAI to examine the link between women’s empowerment and crop output quantity of rural households in Niger and found a positive relationship between these variables.

Against this background, the objective of this study is to assess the effect of women’s empowerment in agriculture on farm productivity change. To pursue this objective, and in line with other studies [8,10,11] in the literature, we use the WEAI and related measures of women’s empowerment, and test their effect on productivity change of Bangladesh rice farms over the crop cultivation period 2011 and 2014. We focus on rice because rice is the largest crop of Bangladesh and crucial for the country’s agricultural development. Rice production accounts for 69% of the total cultivated area in Bangladesh [12], contributes about 70% of the agricultural GDP, and provides 75% of the caloric needs of the nation [13]. We use the Malmquist productivity index to measure productivity change. We further decompose this index into three components: efficiency change, technical change, and scale efficiency change. Then, we use non-parametric bootstrap regression to examine the effect of women’s empowerment, its individual domains, and other control variables on farm productivity change and its decomposition.

We contribute to the existing literature in two ways. First, we assess the role of different measures of women’s empowerment in agriculture on farm productivity change and its components- a dynamic performance indicator by nature. We are aware of no other study of this type, particularly because so many past studies focus on static assessment presumably due to data limitations. Second, we consider the subjective value judgment of the women’s empowerment concept and employ alternative specifications of women’s empowerment to reaffirm the association between women’s empowerment and productivity change and its components. Our research is crucial because it enhances our knowledge about the consequences of promoting women’s empowerment on the farm economic sustainability.

## 2. Literature review

In this section, the relevant literature on productivity change in Bangladesh agriculture is reviewed. The theoretical background of the concept of women’s empowerment is presented and discussed. Finally, the empirical literature that links women’s empowerment in agriculture with agricultural production, nutrition, and food security is examined. In doing so, we focus attention on the most relevant studies rather than the broad literature on women’s empowerment or the long and vast literature on agricultural productivity, including crop yields.

### 2.1 Productivity of Bangladesh agriculture

Growth in agricultural productivity is a fundamental precondition for sustainable economic development, and low agricultural productivity can substantially delay industrialization [14,15]. Agricultural growth is effective in reducing poverty, and it mostly affects the poorest of the poor [16,17]. A few studies have measured productivity change in Bangladesh agriculture, all at the regional level (greater district). Rahman and Salim [18] and Hossain et al. [19] used non-parametric techniques to measure regional productivity change in Bangladesh agriculture and reported a productivity growth of 0.57% and 2.95%, respectively. Coelli et al. [20], on the other hand, employed a parametric approach (stochastic frontier analysis) to measure regional productivity growth in Bangladesh farming and reported a 0.23% decline in productivity. These studies also identified farm size, crop specialization, investment in agricultural research and development, extension contact, and extension expenditure as significant determinants of regional productivity change in Bangladesh. Although there are studies [8,21–24] that used farm-level survey data to analyze farm household technical efficiency until now no studies have measured the changes in farm-level productivity and its components of Bangladesh agriculture.

### 2.2 Women’s empowerment and broader development issues

Because of the subjective nature of the women’s empowerment concept, many definitions have been proposed in the literature. Mehra [25] defined women’s empowerment as the expansion of choices and an increase in women’s ability to exercise these choices. Kabeer [26] defined empowerment as the expansion in people’s ability to make strategic life choices where this ability was previously denied to them. Kabeer’s definition offers an extension to the previous definition in that it indicates a priori requirement for empowerment. The definition indicates that to be empowered, it is necessary to be disempowered first, more specifically, to be in a state where a woman had limited or no freedom of choices, expressions, and actions. Mosedale [27] discussed four characteristics of empowerment: first, the issue of empowerment arises because of the prevalence of disempowerment; second, the urge for empowerment comes from within, it cannot be bestowed by a third party such as government agencies or NGOs; third, being empowered means ability to make and implement decisions regarding aspects that matter most to individuals; and fourth, empowerment is an ongoing process in which people are empowered or disempowered relative to others or themselves in the past. Batliwala [28] further extended the idea and conceptualized empowerment as a socio-political process, where political, social, and economic power shifts between and across both individuals and social groups. All these discussions lead us to two unique defining features of women’s empowerment concept: the process, transformation towards greater freedom of choice, action, and equality; and the agency, woman as an agent of change influences important life outcomes by formulating strategic choices and controlling resources and decisions [29]. For the purpose of this study, we view the empowered woman as an assertive member of a farm household, and the degree of empowerment is contingent upon a woman’s perceived belief in her ability to exercise control over various dimensions of agriculture (e.g., production, resources).

The importance of women’s empowerment as a tool for development can be well approximated by the issue’s inclusion in the United Nation’s Sustainable Development Goals (SDGs), which calls for creating a conducive environment for women where they have higher decision-making power and control over political, economic and public life. Several studies [30,31] echoed this view as they reported that when women have control over cash income, expenditure patterns lean more toward human development inputs such as food and education. Duflo [32] stated that women’s empowerment and economic development are bi-directional in the sense that economic development drives down gender inequality and gender bias hinder development.

One of the most well-known measures of farm households’ women’s empowerment is the index (WEAI) developed by Alkire et al. [9]. WEAI focuses on the agency aspect of the women’s empowerment. It measures the ability of women in farm households to make decisions that relate directly to agriculture. WEAI has been used extensively in various studies that documented the impact of women’s empowerment in agriculture on agricultural production, nutrition, and food security. For example, Sraboni et al. [33] employed WEAI to examine the relationship between women’s empowerment in agriculture and per capita calorie availability, dietary diversity, and adult body mass index (BMI) in Bangladesh. Malapit et al. [34] used WEAI to investigate the relationship between women’s empowerment and dietary diversity and anthropometric outcomes of mothers and children in rural Nepal. Cunningham et al. [35] examined the link between women’s empowerment in agriculture and child nutritional status in rural Nepal. Malapit and Quisumbing [36] used WEAI to investigate the association between women’s empowerment and the nutritional status of women and children in northern Ghana. As discussed in the previous section, some studies [8,10,11] used WEAI to study the linkage between women’s empowerment and technical efficiency or crop output quantity. In conclusion, the literature reviewed indicates an apparent gap in our understanding of the link between women’s empowerment and farm-level productivity change. This study makes an important contribution to the literature by being the first to look at the relationship between women’s empowerment and farm-level productivity and its components.

## 3. Methodology

### 3.1 Conceptual framework

Households can be viewed as a single production unit with a collective structure and members with dissimilar preferences [37,38]. This assumption of cooperative arrangements among members is more consistent as opposed to the unitary model that assumes a single set of household preference and representative altruistic household head [39,40]. Therefore, the relationship between productivity change and women’s empowerment can be conceptualized in terms of a collective bargaining model, where a household shares a stock of resources, and the allocation and use of which is influenced by bargaining power or gendered division of preferences and weights that are being placed on different intrahousehold decisions. Seen in this light, the women’s empowerment in the household corresponds to their say in household decision making and can consequently affect the outcomes, including the decisions that determine the inputs used to generate output. In the context of Bangladeshi rice-producing households, women’s empowerment could affect household productivity change through the effects on the number or size of plots used, the labor applied, or other inputs purchased, but this is speculation in the absence of focused study. Several studies [40–42] indicate that women’s bargaining power positively affects household outcomes such as household food security, girls’ school enrollment, and children’s health status. Higher food security and improved health of the household members could in turn result in a more productive workforce when household members are working at the farm, leading to higher productivity. In another example, if more empowered women are involved in economic or social groups in rural areas, they may bring ideas about new farm practices and technologies that have the potential to increase productivity through, for example, more efficient use of farm resources. In theory, at least, a shift in bargaining power might reallocate resources and effort away from agricultural output and towards other household goals that are given newfound priority, although we are not aware of any empirical finding that would support such a claim. In what follows, the empirical methods used to estimate farm household productivity change and assess the relationship between productivity change and women’s empowerment are presented.

### 3.2. Empirical framework

#### 3.2.1 Measuring productivity change

Following the literature [18,23,43,44], we use the input-oriented Malmquist index to measure farm productivity change. The input orientation is appropriate since farmers have more control over inputs than outputs, which may be affected by external factors such as weather conditions. The input-oriented Malmquist index is based on the input distance function introduced by Shephard [45], which seeks the maximal proportional reduction of an observed input bundle for a given output bundle.

The production technology of a farm household using inputs *x*^*t*^ in period *t* to produce output *y*^*t*^ is represented by the following technology set:

$$T^{t}=\{(x^{t},y^{t}):\;x^{t}\;can\;produce\;y^{t}\}$$ (1)

Another equivalent set-wise characterization of the production technology can be given via the input requirement set of farm households as:

$$L(y^{t})=\{(x^{t}:\;(x^{t},y^{t})\;is\;feasible\}$$ (2)

The input requirement set in (2) represents all possible combinations of inputs that can produce a particular level of output.

A convenient generalization of the production technology, which is a key instrument of efficiency and productivity analysis, is the Shephard’s input distance function. The Shephard’s input distance function for period *t* can be defined as:

$$D_{i}^{t}(x^{t},y^{t})=max\;\{\lambda :\;(x^{t}/\lambda )\in L(y^{t})\},$$ (3)

where *λ* is the value of the input distance function. We can obtain $D_{i}^{t+1}(x^{t+1},y^{t+1})$ by substituting *t* for *t*+1. $D_{i}^{t}(x^{t},y^{t})$ and $D_{i}^{t+1}(x^{t+1},y^{t+1})$ are called single-period distance functions. One can also obtain the following mixed-period distance functions: $D_{i}^{t}(x^{t+1},y^{t+1})$ and $D_{i}^{t+1}(x^{t},y^{t})$. The first of these distance functions concerns the firms at time *t*+1 in relation to the technology at time *t*. The second distance function concerns the firms at time *t* relative to the technology at time *t*+1. Färe et al. [46] used single and mixed-period distance functions to compute the Malmquist productivity index. Assuming a constant return to scale, they defined the input-oriented Malmquist index as:

$$M(x^{t}{{,y}^{t},x}^{t+1}{,y}^{t+1})={\left[\frac{D_{c}^{t}(x^{t+1},y^{t+1})}{D_{c}^{t}(x^{t},y^{t})}\times \frac{D_{c}^{t+1}(x^{t+1},y^{t+1})}{D_{c}^{t+1}(x^{t},y^{t})}\right]}^{1/2},$$ (4)

where *M* is the input-oriented productivity index, which quantifies the changes in productivity originating from the efficiency change measured under period *t* technology and from the efficiency change measured under period *t+1* technology. Following Ray and Desli [47], assuming a variable returns to scale (subscript v stands for VRS and c stands for CRS), we decompose the Malmquist index into efficiency change (EC), technical change (TC), and scale efficiency (SE) change:

$$M(x^{t},y^{t},x^{t+1},y^{t+1})=\underset{EC}{\underset{︸}{\frac{D_{v}^{t+1}(x^{t+1},y^{t+1})}{D_{v}^{t}(x^{t},y^{t})}}}\times \underset{TC}{\underset{︸}{{\left[\frac{D_{v}^{t}(x^{t+1},y^{t+1})}{D_{v}^{t+1}(x^{t+1},y^{t+1})}\times \frac{D_{v}^{t}(x^{t},y^{t})}{D_{v}^{t+1}(x^{t},y^{t})}\right]}^{1/2}}}$$ (5)

$$\begin{array}{l} \times {\underset{SE}{\underset{︸}{\left[\frac{D_{c}^{t+1}(x^{t+1},y^{t+1})/D_{v}^{t+1}(x^{t+1},y^{t+1})}{D_{c}^{t+1}(x^{t},y^{t})/D_{v}^{t+1}(x^{t},y^{t})}\times \frac{D_{c}^{t}(x^{t+1},y^{t+1})/D_{v}^{t}(x^{t+1},y^{t+1})}{D_{c}^{t}(x^{t},y^{t})/D_{v}^{t}(x^{t},y^{t})}\right]}}}^{1/2}\\ \;={EC}_{i}(x^{t},y^{t},x^{t+1},y^{t+1}){\times TC}_{i}(x^{t},y^{t},x^{t+1},y^{t+1})\times {SE}_{i}(x^{t},y^{t},x^{t+1},y^{t+1}) \end{array}$$

EC is the relative efficiency change index which measures catch-up or the degree to which a Decision-Making Unit (DMU) improves or worsens its efficiency relative to the DMUs in the best practice frontier. It reflects a DMU’s ability to use the available knowledge and technology to reach the best practice frontier. TC, often termed interchangeably with technological change, indicates the frontier shift reflecting changes in the efficient frontiers between two time periods. Therefore, TC represents a DMU’s level of innovativeness. SE change measures the closeness of a DMU to its most efficient (optimal) scale size. Technically, it may be interpreted as the relative contraction of inputs by producing at optimal scale on the best practice frontier for the observed input mix of a DMU whose technical inefficiency has been eliminated. Or geometrically, it may be defined as the ray average productivity after the observed mixture of inputs is projected to the best practice frontier relative to what is achievable at the optimal scale [48]. Values of *M*(⋅) or any of its components greater (less) than one implies productivity regress (improvement). Following Odeck [49], for the ease of interpretation, we take the inverse of productivity scores and its components, such that an index greater (less) than one implies productivity improvement (regress).

The distance functions defined above are empirically estimated using Data Envelopment Analysis. More specifically the distance function $D_{i}^{t}(x^{t},y^{t})$ is calculated by solving the following linear problem:

$${\left[D_{i}^{t}(x^{t},y^{t})\right]}^{-1}=\underset{\lambda ,\theta }{\min}\lambda$$

subjectto

$$y_{m0}^{t}\leq \sum_{i=1}^{k}\theta_{i}y_{mi}^{t},\;m=1,2,\ldots ,M$$

$$\lambda x_{n0}^{t}\geq \sum_{i=1}^{k}\;\theta_{i}x_{ni}^{t},\;n=1,2,\ldots ,N$$ (6)

$$\theta_{i}\geq 0,\;s=1,2,\ldots ,S$$

$$\sum_{i=1}^{K}\theta_{i}=1,$$

where *λ* is a scalar that represents the efficiency score for the *i*^th^ household, and *θ* are household weights that define the best practice frontier. In a similar way, $D_{i}^{t+1}(x^{t+1},y^{t+1})$ is computed by substituting *t* for *t*+1.

The mixed period distance function $D_{i}^{t}(x^{t+1},y^{t+1})$ which calculates the efficiency of firms observed in period *t*+1 relative to the period *t* technology is given by:

$${\left[D_{i}^{t}(x^{t+1},y^{t+1})\right]}^{-1}=\underset{\lambda ,\theta }{\min}\lambda$$

subjectto

$$y_{m0}^{t+1}\leq \sum_{i=1}^{k}\theta_{i}y_{mi}^{t},\;m=1,2,\ldots ,M$$ (7)

$$\lambda x_{n0}^{t+1}\geq \sum_{i=1}^{k}\theta_{i}x_{ni}^{t},\;n=1,2,\ldots ,N$$

$$\theta_{i}\geq 0,\;s=1,2,\ldots ,S$$

$$\sum_{i=1}^{K}\theta_{i}=1$$

The mixed period distance function $D_{i}^{t+1}(x^{t},y^{t})$ is obtained analogously.

#### 3.2.2 Modeling the relationship between productivity change and women’s empowerment

After computing the household-specific TFP growth and its components, we use the following model to examine the association between productivity change and women’s empowerment (and a set of control variables):

$$y_{i}=\alpha +z_{i}\beta +u_{i},$$ (8)

where, *y*_*i*_ is the input-oriented Malmquist productivity change (or its components) for household *i*, α is an intercept and *z*_*i*_ is the base period vector of exogenous independent variables that represents women’s empowerment measures, and other control variables for the household *i*, *β* is the vector of coefficients to be estimated and *u*_*i*_ is an error term.

We follow the non-parametric bootstrap method of Kapelko et al. [50], as summarized here. We estimate Eq (8) using an OLS bootstrap regression. A bootstrap regression is required to address the well-known problem of serial correlation among non-parametrically derived productivity and efficiency scores. This approach is a standard non-parametric bootstrap that involves randomly drawing, with replacement, a large number of subsamples from the original sample, and allows the computation of bootstrap regression coefficients and confidence intervals. Our computations involved 5,000 bootstrap replicates. Eq (8) was estimated separately for productivity change and each of its components.

#### 3.2.3 Endogeneity issues

Two endogeneity issues complicate the estimation of the relationship between women’s empowerment and productivity change. The first one is simultaneity or reverse causation. As Wouterse [11] argues, higher empowerment could increase productivity, but at the same time, higher productivity may result in higher income levels, and as a result, enhance household members’ empowerment. However, we believe simultaneity is not an issue in our research setting because we use the base period WEAI as an explanatory variable in Eq (8). Therefore, the initial status of a woman in a household is exogenous with respect to how much productivity changes after the initial condition. The second endogeneity issue is omitted variable bias. Given that the WEAI indicators considered here are perceived measures of a woman’s ability to exercise control over various dimensions of agriculture, there might be unobserved factors determining both the empowerment in the base year and productivity change. To empirically address the potential endogeneity of WEAI variables, a Wu-Hausman test for exogeneity of these variables was conducted [51]. Following Diiro et al. [10], domestic violence, dependency ratio, and age and education differences between the principal male respondent and principal female respondent in the household were used to instrument for the WEAI variables. As it will be discussed in more detail in the results section, the Wu-Hausman test results support the exogeneity of WEAI variables. However, recognizing that the used instruments are unlikely to respect the exclusion restriction and better instruments could not be found, it is appropriate to exercise caution when it comes to attributing causality. As a consequence, in this article we speak of association (or correlation) rather than causation.

## 4. Data

The data used in this study is taken from Bangladesh Integrated Household Survey (BIHS). BIHS is a nationally representative survey in Bangladesh with multiple rounds, of which the 2011/12 and 2015/16 rounds are available at this time (see Ahmed [52] for details). The first round (2011/12) and second round (2015/2016) of data collection relate to crop cultivated during December 2010 to November 2011 and December 2013 to November 2014, respectively By using a two-stage stratified sampling technique and a sampling frame developed from the community series of the 2001 population census of Bangladesh, BIHS collects detailed data on (1) plot-level agricultural production and practices, (2) dietary intake of individual household members, (3) anthropometric measurements (height and weight) of all household members, and (4) WEAI. BIHS includes 6,500 households in 325 Primary Sampling Units (PSUs) or villages. This study focuses on farms engaged primarily in rice production, and for this purpose, we have selected households whose value of total rice production is at least 80% of their total annual farm revenue and participated in both survey rounds. There are 33 households in the dataset that have split between rounds. For comparison purposes, we only considered the original household (parent household) in such cases.

Some variables in the dataset are reported at the plot-level. Other variables, including rice output, capital assets, and women’s empowerment, are reported for each household, which precludes a plot-level analysis. And, fundamentally, our research question is about the association of women’s empowerment and household productivity change, so we do not focus on plot productivity nor, in any case, can we know the variation in women’s empowerment as it relates to individual plots from these survey data. As a result, plot-level information was aggregated to the household-level. Although the survey asks about plots, we cannot track plots between survey rounds. The final dataset consists of a balanced panel of 1,197 households with a total of 2,394 observations. On average, in both rounds five cultivable/arable plots are available to sample farmers. The data needed to compute the input distance functions in Eqs (6) and (7) (that are in turn used to calculate the Malmquist productivity index and its components) include one output and four inputs (Table 1). The output variable includes rice that is produced and either sold or used in the household itself, with the total value of these two uses divided by the consumer price index (CPI)-food [53] to find the total volume as an implicit quantity index. The input variables consist of land, labor, capital and equipment, and miscellaneous input expenses. Land is measured as the total area of cultivable/arable plots (owned or otherwise) used by each household. Labor is the total annual hours of hired and family labor. Capital and equipment are defined as the value of farming tools, machinery, and other tools used in the production process, and is readily available at the household level. Finally, miscellaneous expenses include the value of variable expenses such as seed, irrigation, agrochemicals, and other expenses. All monetary inputs (i.e., capital and miscellaneous inputs) were transformed to implicit quantity indices by calculating the ratio of value to the Bangladesh CPI-Food, with base period being that of 2005/06.

**Table 1:** Summary statistics of the deflated outputs and inputs of the sample households.

**2011**					
**Variables**	**Unit**	**Mean**	**Std. Dev**	**Min**	**Max**
Output	Taka*	26,485	27,803	544	26,7488
Land	Decimal	131	137	4	1,620
Labor	Hours	484	463	30	6,304
Capital and equipment	Taka*	5,287	26,324	5	491,450
Miscellaneous expenses	Taka*	4,798	4,527	207	39,271
**2014**					
**Variables**	**Unit**	**Mean**	**Std. Dev**	**Min**	**Max**
Output	Taka*	24,436	27,117	227	276,017
Land	Decimal	135	145	3	1,665
Labor	Hours	599	581	15	7,435
Capital and equipment	Taka*	5,988	29,720	9	867,542
Miscellaneous expenses	Taka*	7,526	7,571	56	92,308

Source: Authors’ calculations.

Note: The asterisk superscript (*) denotes an implicit quantity index measured in constant 2005/06 prices.

Concerning the data used in the second stage of our analysis, the *z*_*i*_ vector in Eq (8) includes a household-specific women’s empowerment measure (e.g., women’s empowerment score, gender parity gap, individual empowerment domains, or empowerment indicators), and a set of control variables. The following subsections describe the procedure followed to calculate the different women’s empowerment measures for each household and the variables included in the control variables.

### 4.1 Women’s empowerment in agriculture measures

The women’s empowerment in agriculture measures used in this study are based on the women’s empowerment in agriculture index (WEAI) developed by Alkire et al. [9]. WEAI is a survey-based index that measures empowerment, agency, and inclusion of women in agriculture. WEAI is an aggregate measure with two sub-indices namely five domains of empowerment (5DE) and gender parity index (GPI).

The first index, 5DE (empowerment score thereafter), is an estimation of respondents’ empowerment score based on their role in five domains of agricultural decision making (Table 2). An individual-level questionnaire (see Alkire et al. [54] for details) is used as the primary instrument to measure empowerment using weighted domain-specific indicators. For example, group membership indicator, of the leadership domain, is given a weight of 0.10, and this weight relates to questions about membership in economic or social groups. If the respondent reports membership in at least one economic or social group, she scores 0.10 under this indicator. The empowerment score is calculated similarly for the other nine indicators, and a composite empowerment score is then generated by taking the weighted sum of all ten indicators. By construction, the empowerment score ranges from 0 to 1. Alkire et al. [9] judge that a score of 0.80 or more in the composite scale is required to deem a woman as empowered. The second index, GPI, is the relative measure of inequality and estimated using the difference between 5DE scores of each household’s primary adult male and female. The gender parity gap (empowerment gap thereafter) takes a value of zero if the primary female decision maker’s 5DE score exceeds or is equal to that of primary male decision maker. This measure reflects the relative empowerment of the women compared to the men in decision making. Gender parity is achieved when the empowerment score of the primary female meets or exceeds the empowerment score of the primary male living in the same household.

**Table 2:** WEAI domains.

Domain	Indicator	Description	Weight
Production	Input in productive decisions	Ability to make decisions (sole or joint) about food and cash-crop farming, livestock, and fisheries	1/10
	Autonomy in production	Ability to act according to own value and judgment regarding inputs to buy, types of crops to grow, when to take or who would take crops to market, and livestock production	1/10
Resources	Ownership of assets	Sole or joint ownership of household assets such as land, livestock, consumer durables, agricultural equipment	1/15
	Purchase, sale, or transfer of assets	Decision-making authority over the purchase, sale, or transfer of household assets	1/15
	Access to and decisions about credit	Decision-making authority over obtaining credit and using credit proceeds	1/15
Income	Control over the use of income	Sole or joint control over income and expenditures	1/5
Leadership	Group membership	Active membership in at least one economic or social group	1/10
	Speaking in public	Ability to speak up in public for reasons like to ensure proper payment of wages for public work programs, to protest the misbehavior of authorities or to help decide on infrastructure	1/10
Time	Workload	The productive and domestic workload in a 24-hour framework	1/10
	Leisure	Subjective satisfaction with available leisure time	1/10

Source: Alkire et al. [9].

WEAI operates under predetermined assumptions to capture the power dynamics of the farm household’s women. Such dynamics may alter under much wider or restrictive assumptions. For example, under the leisure indicator, a woman is deemed as empowered if she is at least neither satisfied nor dissatisfied with the time available for leisure. One might argue that such a definition of empowerment (related to leisure time) is not the true depiction of empirical reality because the assumption does not distinguish between satisfaction and dissatisfaction with available leisure time, nor does it reward a reported higher level of satisfaction with a larger scale. Such variation in the subjective assessment of empowerment will result in different estimates of empowerment score. We capitalized on this idea and developed two alternative empowerment scoring approaches by modifying the WEAI assumptions to refocus this variable on our specific requirements (see online supplement S1 in S1 File for more details). A summary of the alternative measures used to represent women’s empowerment in agriculture is given in Table 3.

**Table 3:** Summary statistics of WEAI and alternative empowerment measures.

	*WEAI- Based on Alkire et al*. [9]				*Alternative approach 1*				*Alternative approach 2*			
	Mean	Std. dev	Min	Max	Mean	Std. dev	Min	Max	Mean	Std. dev	Min	Max
Empowerment score	0.60	0.19	0.10	1.00	0.30	0.12	0.03	0.72	0.53	0.16	0.09	0.97
Empowerment gap	0.15	0.17	0.00	0.80	0.10	0.11	0.00	0.62	0.14	0.15	0.00	0.82
Domains												
Production	0.10	0.07	0.00	0.20	0.07	0.06	0.00	0.20	0.11	0.06	0.00	0.20
Resources	0.13	0.06	0.00	0.20	0.03	0.02	0.00	0.11	0.11	0.06	0.00	0.20
Income	0.15	0.08	0.00	0.20	0.05	0.04	0.00	0.20	0.12	0.06	0.00	0.20
Leadership	0.06	0.07	0.00	0.20	0.01	0.02	0.00	0.11	0.04	0.05	0.00	0.20
Time	0.15	0.06	0.00	0.20	0.12	0.06	0.00	0.20	0.15	0.06	0.00	0.20

Source: Authors’ calculations.

Under the first alternative approach, we developed empowerment scores based on the gradations of women’s power rather than binary assessments. The second alternative approach focuses on a woman’s engagement and relative input in the activity or decision-making process in which a household participates. For each of these approaches, scores of empowerment, gender parity gap, individual empowerment domains, and indicators were calculated. The objective behind the formulation of alternatives scores is to improve the tests for an association between women’s empowerment and the productivity change of rice farms in Bangladesh. We can affirm our findings as robust and valid only when the underlying effect estimates remain consistent under different subjective ratings of the empowerment concept. We posit that construction of alternative indices using a single standardized framework that solely focus on agency aspect of women’s empowerment runs the risk of ignoring indirect indicators such as resources and achievements. However, the design and implementation of indicators of women’s empowerment is beyond the scope of this study. Hence, we followed the framework suggested by Alkire et al. [9] and recalculate the index by testing different assumptions regarding the adequacy requirements. Despite the shortcomings, such assumptions allow us to capture different contexts that different households may encounter and measure multidimensional characteristics of women’s empowerment at various levels of aggregation.

### 4.2 Control variables

The selection of control variables included in Eq (8) was guided by data availability and previous studies on the determinants of farm productivity [5,18,20]. These variables include sex, age, education, dependency ration, household size, extension visit, rainfed, tenancy, income share from non-agricultural enterprise, rainfall, temperature, and locational dummies (southeast, northeast, southwest, and northeast). Table 4 presents the definition and summary statistics of these variables.

**Table 4:** Control variables and descriptive statistics.

Variables	Description	Mean	Std. dev	Min	Max
Sex	Categorical: one if female else zero	0.02	0.14	0.00	1.00
Age	Primary respondent’s age	46.37	12.46	18.00	85.00
Education	Years of education completed by the primary respondent	3.23	3.92	0.00	16.00
Dependency ratio	The ratio of children (0–14 years old) and nonworking-age household members (65 years or older) to working-age household members (15–64 years old)	0.76	0.60	0.00	5.00
Household size	Total members in the household	4.69	1.68	2.00	14.00
Extension visit	Number of visits by an agricultural extension agent to the household or by a household member to an extension service office during the last 12 months	0.35	1.32	0.00	15.00
Rainfed	Categorical: one if rain is the primary source of water for cultivation else zero	0.37	0.48	0.00	1.00
Tenancy	Categorical: one if the land is taken-in through a cash lease or crop-sharing arrangement else zero	0.33	0.47	0.00	1.00
Non-agric. income share	Share of household income from non-agricultural sources	0.22	0.31	0.00	1.00
Rainfall	Difference between the average annual rainfall of crop cultivation years 2014 and 2011 (in millimeters)	-224.99	299.31	-487.00	349.00
Temperature	Difference between the average of minimum and maximum temperature of crop cultivation year 2014 and 2011 (in Celsius)	0.43	0.20	0.20	0.80
Northwest	Categorical: one if northwest divisions else zero	0.20	0.40	0.00	1.00
Southwest	Categorical: one if southwest divisions else zero	0.23	0.42	0.00	1.00
Northeast	Categorical: one if northeast divisions else zero	0.15	0.36	0.00	1.00

Note: For household with more than one plot, the rainfed dummy gets the value 1 if rain was the primary irrigation source in the majority of the plots. A similar rule applies for the tenancy status variable; Rainfall and temperature data are collected from the yearbook of agricultural statistics -2014 [55].

Source: Authors’ calculations.

## 5. Results

The Malmquist productivity index and its decomposition are summarized in Table 5. Before discussing these results, it should be noted that the mixed-period linear programs used to calculate the Malmquist productivity index and its components may give infeasible solutions. A possible remedy to this problem is to exclude the observations with infeasible solutions from the calculation of average productivity change and its components [50]. Briec and Kerstens [56] argue that studies computing productivity indices using non-parametric estimators should report the infeasibilities resulting from the estimation of the mixed-period distance functions. In our case, out of 1,197 observations, only one observation has an infeasible solution for one of the mixed period distance functions. This observation was excluded in the second stage analysis.

**Table 5:** Average Malmquist productivity index and its components of the sample households for the period 2011 and 2014.

	Productivity change	Efficiency change	Technical change	Scale efficiency change
Mean	0.76	1.15	0.65	1.03
Std. Dev.	0.40	0.57	0.10	0.21
Min	0.12	0.13	0.29	0.62
Max	3.78	4.35	2.10	6.93
95% Conf. Interval				
Lower bound	0.73	1.12	0.64	1.02
Upper bound	0.78	1.18	0.65	1.04

Source: Authors’ calculations.

Over the estimation period, on average, productivity fell by 24%, technical efficiency grew by 15%, technological change fell by 35%, and scale efficiency grew by 3%. Putting these results in perspective with that of other studies from Bangladesh agriculture, we see that our estimate of productivity decrease is more severe than that of Coelli et al. [20], who reported a 0.23% decline in productivity, and contrasts with increases reported by Hossain et al. [19], at 2.95%, and Rahman and Salim [18], at 0.57%. However, the findings of this study are not directly comparable to those studies due to the difference in the sample. This study uses plot-level data (aggregated to household level), whereas the aforementioned studies used regional level data. Other notable differences include crop type analyzed (e.g., Coelli et al. [20] aggregated food and cash crops) and estimation method employed (e.g., Rahman and Salim [18] used the Färe-Primont index to measure productivity).

Decomposition results indicate that productivity in Bangladesh rice farming fell due entirely to the decline in technical change. Shifts in technical change can be considered to be evidence of innovation [57]. In the simplest analysis, lack of innovation in the current production technology or the adoption of a production technology that is not innovative enough to reflect the needs of the farmers might cause a decline in technical change, which in turn contributes to overall productivity decline. One potential cause of technological regress could be declining soil quality, with evidence suggesting that cropping practices reduce soil organic matter with negative impacts on Bangladesh rice production [58–60].

In the second stage, we estimated several specifications of Eq (8) to analyze the relationship between productivity indices and the different women’s empowerment measures. Before presenting the results of the determinants of productivity change and its components, we first discuss the results of the Wu-Hausman test of the exogeneity of the WEAI variables. The results of the exogeneity tests (results are available from the authors upon request) indicate that the null hypothesis of exogeneity of the WEAI variables cannot be rejected in all specifications. However as noted earlier and given the absence of better instruments in our database, the WEAI results will be interpreted as implying correlation rather than causation.

Columns 2–5 of Table 6 present the bootstrap regression estimates of the impact of women’s empowerment (empowerment score based on Alkire et al. [9]) and other control variables on productivity change and its decompositions. The results show that except for scale efficiency change, the relationship between women’s empowerment in agriculture and productivity change, efficiency change, and technical change is positive and statistically significant at 1%, 10%, and 5% level, respectively. Columns 6–13 of Table 6 further reinforce this association; alternative specifications of women’s empowerment scores are also statistically significant at 1% and 5% level, and positively related to the Bangladesh rice farming productivity change, efficiency change, and technical change.

**Table 6:** Results of the OLS bootstrap regression of the determinants of farm productivity change and its components.

	Model 1: Empowerment score based on Alkire et al. [9]				Model 2: Alternative empowerment score based on approach 1				Model 2: Alternative empowerment score based on approach 2			
Variables	Productivity change	Efficiency change	Technical change	Scale efficiency change	Productivity change	Efficiency change	Technical change	Scale efficiency change	Productivity change	Efficiency change	Technical change	Scale efficiency change
Empowerment score	0.169***	0.166*	0.038**	-0.017	0.438***	0.460***	0.075***	0.041	0.251***	0.292***	0.038**	-0.018
	(0.060)	(0.088)	(0.017)	(0.024)	(0.111)	(0.157)	(0.024)	(0.063)	(0.079)	(0.112)	(0.019)	(0.027)
Sex	-0.107	-0.096	-0.037*	0.019	-0.128*	-0.119	-0.039**	0.015	-0.124*	-0.119	-0.038*	0.020
	(0.070)	(0.108)	(0.020)	(0.025)	(0.071)	(0.112)	(0.019)	(0.026)	(0.069)	(0.108)	(0.020)	(0.026)
Age	0.001	0.001	0.000	0.000	0.001	0.001	0.000	0.000	0.001	0.001	0.000	0.000
	(0.001)	(0.001)	(0.000)	(0.000)	(0.001)	(0.001)	(0.000)	(0.000)	(0.001)	(0.001)	(0.000)	(0.000)
Education	0.001	-0.004	0.001*	0.004*	0.000	-0.005	0.001*	0.004*	0.001	-0.005	0.001*	0.004*
	(0.003)	(0.005)	(0.001)	(0.002)	(0.003)	(0.005)	(0.001)	(0.002)	(0.003)	(0.005)	(0.001)	(0.002)
Dependency ratio	-0.047**	-0.055**	-0.007	-0.006	-0.045**	-0.052*	-0.007	-0.006	-0.046**	-0.054*	-0.007	-0.006
	(0.019)	(0.028)	(0.005)	(0.011)	(0.019)	(0.028)	(0.005)	(0.011)	(0.019)	(0.028)	(0.005)	(0.011)
Household size	-0.014	-0.019*	0.001	-0.005**	-0.013	-0.019*	0.001	-0.005**	-0.013	-0.019*	0.001	-0.005**
	(0.009)	(0.011)	(0.002)	(0.003)	(0.009)	(0.012)	(0.002)	(0.002)	(0.009)	(0.011)	(0.003)	(0.002)
Extension visit	0.013	-0.004	0.007	0.004	0.013	-0.004	0.007	0.004	0.013	-0.004	0.007	0.004
	(0.021)	(0.013)	(0.009)	(0.004)	(0.021)	(0.013)	(0.010)	(0.004)	(0.021)	(0.013)	(0.010)	(0.004)
Income share from non ag. enterprise	0.050	0.111**	-0.011	-0.010	0.048	0.108*	-0.011	-0.011	0.051	0.111**	-0.010	-0.010
	(0.037)	(0.055)	(0.009)	(0.017)	(0.036)	(0.055)	(0.009)	(0.018)	(0.037)	(0.055)	(0.009)	(0.018)
Rainfed	0.020	0.026	0.001	-0.004	0.027	0.033	0.002	-0.004	0.022	0.028	0.001	-0.004
	(0.025)	(0.035)	(0.006)	(0.012)	(0.024)	(0.035)	(0.006)	(0.011)	(0.025)	(0.035)	(0.006)	(0.012)
Tenancy	0.060**	0.059	0.012**	0.011	0.058**	0.057	0.012**	0.011	0.059**	0.057	0.012**	0.011
	(0.026)	(0.037)	(0.005)	(0.017)	(0.025)	(0.037)	(0.005)	(0.017)	(0.026)	(0.037)	(0.005)	(0.017)
Rainfall	-0.000*	-0.000	-0.000***	0.000*	-0.000	-0.000	-0.000***	0.000*	-0.000*	-0.000	-0.000***	0.000*
	(0.000)	(0.000)	(0.000)	(0.000)	(0.000)	(0.000)	(0.000)	(0.000)	(0.000)	(0.000)	(0.000)	(0.000)
Temperature	0.261***	0.237*	0.073***	-0.010	0.258***	0.236*	0.071***	-0.003	0.262***	0.244*	0.070***	-0.009
	(0.091)	(0.135)	(0.024)	(0.027)	(0.089)	(0.133)	(0.023)	(0.025)	(0.090)	(0.134)	(0.024)	(0.026)
Northwest	0.041	-0.011	0.026***	0.034	0.054	0.004	0.028***	0.037	0.045	-0.004	0.026***	0.034
	(0.034)	(0.047)	(0.008)	(0.024)	(0.033)	(0.048)	(0.008)	(0.026)	(0.034)	(0.048)	(0.008)	(0.024)
Southwest	-0.043	0.003	-0.028***	-0.000	-0.025	0.023	-0.025**	0.003	-0.039	0.010	-0.028***	-0.000
	(0.038)	(0.054)	(0.010)	(0.010)	(0.037)	(0.054)	(0.011)	(0.011)	(0.038)	(0.053)	(0.011)	(0.010)
Northeast	0.171***	0.157*	0.054***	-0.025	0.175***	0.162*	0.054***	-0.022	0.177***	0.165*	0.054***	-0.025
	(0.059)	(0.087)	(0.015)	(0.022)	(0.058)	(0.087)	(0.015)	(0.022)	(0.057)	(0.088)	(0.015)	(0.022)
Constant	0.510***	0.977***	0.556***	1.063***	0.480***	0.937***	0.557***	1.037***	0.476***	0.915***	0.559***	1.062***
	(0.092)	(0.136)	(0.024)	(0.037)	(0.089)	(0.130)	(0.022)	(0.032)	(0.095)	(0.137)	(0.024)	(0.036)

Note: Standard errors in parentheses

*, **, and *** indicate significance based on 90%, 95% and 99% bootstrap confidence level, respectively.

The positive relationship between empowerment scores and productivity change indicates that a 1% increase in women’s empowerment score is associated with farm productivity increases of 0.17% to 0.44%. We can also infer that farm productivity increases by 3% to 22% when the primary female decision maker of the household achieves adequacy in at least 80% of the weighted indicators (threshold level empowerment). We calculate this by multiplying the estimated parameter with the average gap between the 80% index threshold required to be considered empowered and the average empowerment score. The results also indicate that empowering farm household women can move a farm household toward the best practice frontier. A 1% increase in empowerment score implies 0.17% to 0.46% increase in efficiency change or, alternatively, if the primary female decision maker achieves an adequacy score of 0.80, the efficiency change index improves by 3% to 23%. This indicates that women’s greater agency over WEAI domains has the potential to help farm households to increase the level of utilization of the potential (maximal capacity) of the production technology. Similarly, findings also indicate that empowering woman shifts farm’s production frontier upward by 0.04% to 0.08%. This implies that lifting primary female decision maker’s empowerment score up to the threshold level of empowerment could improve the technical change index by 1% to 4%. This indicates that there is scope for the sample households to increase technological levels and innovative capabilities through ensuring farm household women’s agency over domains of WEAI.

Table 6 (Columns 2–5) also shows that, among the household-level characteristics, sex, education, dependency ratio, household size, and income share from the non-agricultural enterprise have statistically significant effects on the productivity indices (for a detailed explanation of control variables see online supplement S2 in S2 File).

Table 7 reports the effect of empowerment gap on productivity indices, estimated using the approach detailed in section 3.2 (see online supplement S3 in S3 File for the full set of results). The results (column 2) indicate that the empowerment gap is negatively associated with productivity change and this effect is statistically significant at the 5% level. Empowerment gap calculated based on alternative approach 1 (column 3) also has a significant negative impact on productivity change, further reiterating the findings of column 2. This implies that a 1% reduction in the gap between primary male and female decision maker’s 5DE score is associated with an increase in farm household’s overall productivity by 0.13% to 0.18%. The results of the control variables (see online supplement S3 in S3 File) are similar to those reported in Table 6 in terms of signs and significance, and the magnitudes of the coefficients are also quite similar.

**Table 7:** Results of the OLS bootstrap regression of the determinants of farm productivity change and its components (Empowerment gap).

Dependent variable	Model 1: Empowerment gap based on Alkire et al. [9]	Model 2: Alternative empowerment gap based on approach 1	Model 3: Alternative empowerment gap based on approach 2
Productivity change	-0.128**	-0.182*	-0.117
	(0.063)	(0.109)	(0.077)
Efficiency change	-0.105	-0.224	-0.123
	(0.094)	(0.157)	(0.112)
Technical change	-0.027	-0.016	-0.013
	(0.018)	(0.028)	(0.021)
Scale efficiency change	0.002	-0.007	-0.001
	(0.030)	(0.054)	(0.033)

Note: Standard errors in parentheses

*, **, and *** indicate significance based on 90%, 95% and 99% bootstrap confidence level, respectively.

Table 8 reports the effect of WEAI domains on productivity indices (see online supplement S3 in S3 File for the full set of results). Results indicate that farm household women’s agency over production domain is positively associated with productivity change, efficiency change, and technical change. Findings suggest that a 1% increase in women’s empowerment in production domain leads to a 0.48% to 0.80% increase in productivity of the sample farms. Results also indicate that a 1% increase in primary female decision makers empowerment in production domain leads to efficiency gains of between 0.46% and 0.89%. Empowerment in the production domain is also positively associated with technical change as the results reveal that it can lead to technological gains of about 0.12% In the main model (Model 1), the rest of the WEAI domains were not found to have a statistically significant effect on productivity change and its components, except for the leadership domain which is weakly significant only in one out of the three models tested.

**Table 8:** Results of the OLS bootstrap regression of the determinants of farm productivity change and its components (WEAI domains).

	Productivity change	Efficiency change	Technical change	Scale efficiency change
**Model 1: Empowerment domain score based on Alkire et al.** [9]				
Empowerment score: Production	0.483***	0.458*	0.115***	0.038
	(0.168)	(0.241)	(0.041)	(0.090)
Empowerment score: Resource	0.064	0.091	0.041	-0.067
	(0.179)	(0.265)	(0.042)	(0.058)
Empowerment score: Income	-0.010	-0.075	-0.021	0.026
	(0.140)	(0.211)	(0.034)	(0.044)
Empowerment score: Leadership	0.096	0.224	0.033	-0.196*
	(0.205)	(0.275)	(0.053)	(0.103)
Empowerment score: Time	0.196	0.174	0.016	0.083
	(0.172)	(0.258)	(0.042)	(0.078)
**Model 2: Alternative empowerment domain score based on approach 1**				
Empowerment score: Production	0.798***	0.885***	0.121**	0.147
	(0.233)	(0.329)	(0.054)	(0.179)
Empowerment score: Resource	0.843	0.919	-0.080	0.677
	(0.593)	(0.837)	(0.142)	(0.542)
Empowerment score: Income	-0.091	-0.255	0.124	-0.311
	(0.413)	(0.583)	(0.083)	(0.247)
Empowerment score: Leadership	0.314	0.576	0.047	-0.472
	(0.574)	(0.806)	(0.118)	(0.348)
Empowerment score: Time	0.253	0.293	0.008	0.112
	(0.205)	(0.298)	(0.049)	(0.119)
**Model 3: Alternative empowerment domain score based on approach 2**				
Empowerment score: Production	0.762***	0.838***	0.120**	0.132
	(0.217)	(0.314)	(0.049)	(0.174)
Empowerment score: Resource	0.412**	0.500*	0.047	0.014
	(0.199)	(0.303)	(0.049)	(0.084)
Empowerment score: Income	-0.101	-0.181	0.014	-0.132
	(0.239)	(0.336)	(0.054)	(0.124)
Empowerment score: Leadership	-0.087	0.092	-0.024	-0.217
	(0.249)	(0.330)	(0.064)	(0.133)
Empowerment score: Time	0.187	0.167	0.017	0.077
	(0.171)	(0.255)	(0.042)	(0.080)

Note: Standard errors in parentheses

*, **, and *** indicate significance based on 90%, 95% and 99% bootstrap confidence level, respectively.

We also estimate the effect of indicators of production domain on productivity indices. Results indicate that autonomy in production is statistically significant in all three models and positively associated with productivity change, efficiency change, and technical change (Table 9, also see online supplement S3 in S3 File for the full set of results). This implies that improvements in primary female decision marker’s ability to make independent choices regarding agricultural production increases farm household’s overall productivity through efficiency and technological gains. Input in production decisions, however, is statistically significant in alternative model (Model 2), and positively associated with only technical change.

**Table 9:** Results of the OLS bootstrap regression of the determinants of farm productivity change and its components (production domain indicators).

	Productivity change	Efficiency change	Technical change	Scale efficiency change
**Model 1: Production domain indicator based on Alkire et al.** [9]				
Input in productive decisions	0.045	-0.220	0.087	-0.017
	(0.237)	(0.351)	(0.064)	(0.087)
Autonomy in production	0.944***	1.121***	0.133**	0.094
	(0.239)	(0.338)	(0.056)	(0.130)
**Model 2: Alternative production domain indicator based on approach 1**				
Input in productive decisions	0.564	-0.102	0.390***	0.040
	(0.712)	(0.993)	(0.144)	(0.308)
Autonomy in production	0.896***	1.101***	0.107*	0.088
	(0.240)	(0.345)	(0.057)	(0.120)
**Model 3: Alternative production domain indicator based on approach 2**				
Input in productive decisions	0.175	-0.058	0.071	0.093
	(0.484)	(0.713)	(0.113)	(0.269)
Autonomy in production	0.945***	1.093***	0.143**	0.089
	(0.230)	(0.333)	(0.056)	(0.129)

Note: Standard errors in parentheses

*, **, and *** indicate significance based on 90%, 95% and 99% bootstrap confidence level, respectively.

## 6. Conclusions and discussion

This study contributes to the existing literature with an assessment of the association between women’s empowerment in agriculture and farm household productivity change and its components. This assessment involves two steps. First, a non-parametric Malmquist approach is used to estimate household-specific productivity change and its decomposition. Next, we employ a non-parametric bootstrap OLS regression method to analyze the link between women’s empowerment in agriculture (and a set of control variables) and productivity change and its decomposition. To better understand the relationship between women’s empowerment and productivity change, we also analyze the effect of gender parity gap, individual domains and indicators of women’s empowerment, and the same measures derived using two alternative scoring procedures. The empirical application focuses on Bangladesh rice farms.

Our first-stage results show that over the sample period, farm household productivity decreased by an average of 24%. The main driver of productivity decline was technological regress estimated at 35%. Despite the observed negative technical change, we find that the sample households, on average, used the existing production technology more efficiently over time as we record a 15% improvement in efficiency change during the estimation period. In addition, these households achieved adjustments in production scale that allowed them to obtain a 3% growth in productivity over time. Our second stage results indicate that empowering women in agriculture, specifically improving primary female decision makers ability to make independent choices regarding agricultural production, is positively and significantly associated with productivity change, efficiency change, and technical change, while it is not significantly associated with scale efficiency change. We further found that closing the gender parity gap is associated with higher farm productivity. These results hold for alternative measures of empowerment that we test, which validate the inference that might be drawn from this study. We also find that apart from women’s empowerment in agriculture, climatic variables, and household characteristics, such as sex, education, household size and location, explain differences in productivity change and its components among households in this study.

Besides the intrinsic value of empowering women involved in farming, our main finding, that women’s empowerment in agriculture is associated with higher farm productivity, is even more compelling in view of the fact that population growth, land scarcity, and other resource constraints challenge agricultural regulators and farmers to find innovative ways to use existing farm resources more efficiently. In the case of Bangladesh, women’s empowerment in agriculture might not only be valued for contributing towards development goals, but also as a means for increasing agricultural productivity.

Farm household women’s contribution to agriculture in Bangladesh are traditionally confined to homestead production (farming carried out around homestead) and post-harvest activities such as drying, sorting, packaging of crops [61,62]. Since the interaction pathway between empowerment and productivity is entirely through the production domain, interventions that increase the primary female decision makers relative autonomy in agricultural productive decisions might help the country boost its agricultural productivity.

This research on the relationship between women’s empowerment in agriculture and farm productivity growth focuses on a single developing country. Future research could extend the work in different ways, such as by examining whether similar findings would be observed in different developing countries where cultural and legal (e.g., women’s rights) differences can affect women’s involvement in agricultural decision-making, different time periods or types of farms, and the role of actual policies to improve women’s empowerment where relevant data are available. If sufficient data were available, then the association of women’s empowerment and productivity might be decomposed by growing seasons, crop varieties, production technology heterogeneity, market orientation, or at the scale of field or plot. Exploration into these areas could develop further the empirical association of women’s empowerment and rice farming household productivity change in Bangladesh.

## Supporting information

S1 File Online supplement S1. (DOCX)

S2 File Online supplement S2. (DOCX)

S3 File Online supplement S3. (DOCX)
