# Supplementary material for: Women’s empowerment in agriculture and productivity change: The case of Bangladesh rice farms
Source: PLoS One. 2021 Aug 4;16(8):e0255589. doi: 10.1371/journal.pone.0255589 (PMC8336850; doi:10.1371/journal.pone.0255589)
Supplement: S1 File — (DOCX) [file pone.0255589.s001.docx]

**Online supplement S1**

*Alternative approach 1*

The original WEAI has an adequacy requirement for each indicator. For example, for a woman to be empowered under resources domain, it is adequate for her (i) to own any assets provided that it is not just one small asset (a chicken, nonmechanized equipment or small consumer durables), (ii) to participate in at least one type of decision to buy, sell or transfer a major asset that a household owns and (iii) to contribute to at least one decision regarding credit access and use. We modified these assumptions to develop empowerment scores based on the gradations of women’s power rather than binary assessments. For example, in the resource domain of the first alternative, we assumed that the more assets a woman owns, the higher the empowerment score, and consequently, we record a higher indicator relating to ownership of assets. Similarly, a woman scores higher under the resource domain if she participates in more decisions regarding purchase, sale, or transfer of assets with more significant input compared to a woman who participates in the decision-making process with low input or does not participate at all. The same assumption is employed for access to and decisions about credit: the more access she has and the higher input she provides in decision making regarding sources of credit and credit proceeds utilization, the higher the empowerment score in this indicator.

*Alternative approach 2*

The second alternative approach focuses on a woman’s engagement and relative input in the activity or decision-making process in which a household participates. For example, under the input in productive decision indicator, a woman is asked about her input in productive decisions around activities such as food crop farming, cash farming, livestock raising, non-farm economic activities, wage and salary employment, and fishing or fish culture. The answer scale ranges from 1= “no input” to 5= “input in all decisions.” Under the assumptions of original WEAI, a woman is deemed as empowered if she participates in at least two activities in which she has at least “some input.” The fundamental idea behind the second alternative approach is recognizing that a woman might belong to a household that is engaged in only a specific activity, such as cash crop farming. Alternatively, the household might be engaged in all the activities, but the primary female decision-maker has a varying degree of engagement in those activities. Our idea is to develop a scoring system that does not reduce the empowerment of a woman for not contributing to an activity where a household does not participate and not to exaggerate the empowerment a woman who contributes some input in decision making in a minority of several household pursuits compared to a woman who provides more input into a range of agricultural production decisions.

Assumptions of original WEAI, first and second alternative approach are discussed in detail in Table 1.

Table 1: *Empowerment assumptions*

| **Domains\Index** | **Indicator** | **Weight** | **Survey question** | **WEAI** | ***Alternative approach 1*** | ***Alternative approach 2*** |
| --- | --- | --- | --- | --- | --- | --- |
| **Agricultural Production** | *Input in productive decisions* | 0.10 | How much input did you have in making decisions about food crop farming, cash crop farming, livestock raising, and fish culture? To what extent do you feel you can make your own personal decisions regarding these aspects of household life if you want(ed) to: agriculture production, which inputs to buy, which types of crops to grow for agricultural production, when to take or who should take crops to market, and livestock raising? | Achievement in two activities. Inadequate if the individual participates but does not/has not at least some input in decisions or does not make decisions nor feels he or she could. | No minimum requirement of activities. Both actual inputs and intent are considered. Inputs and extent account for 60% and 40% of the weight, respectively. Weights are assigned based on the answer scale. Adequacy is calculated as the weighted average of every activity and intent. | No minimum requirement of activities. Considers only those activities or intents in which responses have been recorded.  The score is calculated as the weighted average of the degree of the inputs OR intent (cut off is small extent) of activities, whichever is greater. |
|  | *Autonomy in production* | 0.10 | My actions in [domain] are partly because I will get in trouble with someone if I act differently. Regarding [domain] I do what I do so others don’t think poorly of me. Regarding [domain] I do what I do because I personally think it is the right thing to do. Agricultural production, inputs to buy, crops to grow, take to market, livestock. | Achievement in any. Inadequate if Relative Autonomy Indicator (RAI) is less than 1. | Achievement in any. Inadequate if RAI is less than 1. | Achievement in any. Inadequate if RAI is less than 1. |
| **Access to and Control of Productive Resources** | *Asset ownership* | 0.07 | Who would you say can use the [item] most of the time? Agricultural land, large livestock, small livestock, chickens and so on; fishpond/equipment; farm equipment (nonmechanized); farm equipment (mechanized); nonfarm business equipment, house; large durables; small durables; cell phone; nonagricultural land (any); transport. | Achievement in any if not only  one small asset (chickens,  nonmechanized equipment and  no small consumer durables). Inadequate if the household does not own any asset or if the household owns the type of asset but she or  he does not own most of it alone. | Adequacy is calculated as the weighted average of ALL large and small assets owned by self or jointly. | No adequacy requirement. The score is calculated as the weighted average of the assets owned by self or jointly; large assets get twice the weight of small assets. |
|  | *Decisions regarding the*  *purchase, sale, or transfer of land and assets* | 0.07 | Who would you say can decide whether to sell, give away, rent/mortgage [item] most of the time? Who contributes most to decisions regarding a new purchase of [item]? Agricultural land, large livestock, small livestock, chickens and so on, fishpond, farm equipment (nonmechanized), farm equipment (mechanized). | Achievement in any if not only  chickens and nonmechanized  farming equipment. Inadequate if a household does not own any asset or household owns the type of asset but he or she does not participate in decisions (exchange or buy) about it. | Adequacy is calculated as the weighted average of ALL the decisions associated with ALL the assets owned by self or jointly. | Adequacy is calculated as the weighted average of the decisions associated with large and small assets owned by self or jointly; 80% of the weight is assigned to large assets and participating in at least one decision regarding large asset is required. |
|  | *Access to and decisions about credit* | 0.07 | Who made the decision to borrow/what to do with money/item borrowed from [source]? Nongovernmental  organization, informal lender, formal lender (bank), friends or relatives, rotating savings and credit associations (savings/credit group). | Achievement in any. Inadequate if the household has no credit or used a source of credit but she or he did not participate in any decisions about it. | Adequacy is calculated as the weighted average of all the decisions (to borrow and use) regarding all the sources of credit. | At least one source of credit is required. Adequacy is calculated as the weighted average of all the decisions regarding a source in which she participated. |
| **Income** | *Control over use of income* | 0.20 | How much input did you have in decisions about the use of income generated from food crop, cash crop, livestock, nonfarm activities, wages and salary, and fish culture? To what extent do you feel you can make your own personal decisions regarding these aspects of household life if you want(ed) to: your own wage or salary employment? Minor household expenditures? | Achievement in any if not only  minor household expenditures. Inadequate if the individual participates in the activity but has no input or little input in decisions about the income generated. | Adequacy is calculated as the weighted average of the degree of inputs and the extent the respondent feels she can make decisions. Inputs and extent account for 90% and 10% of the weight respectively. | Adequacy is calculated as the weighted average of the degree of inputs or extent the respondent feels she can make decisions, whichever is greater. Input in only minor household expenditures is not enough. |
| **Leadership** | *Group membership* | 0.10 | Are you a member of any agricultural/livestock/fisheries producer/market group; water, forest users’, credit, or microfinance group; mutual help or insurance group (including burial societies); trade and business association; civic/charitable group; local government; religious group; other women’s group; other group? | Achievement in any. Inadequate if not part of at least one group. | Adequacy is calculated as the weighted average of ALL THE MEMBERSHIP OPTIONS (active/inactive) in all eleven groups. | Achievement in any. Inadequate if not part of at least one group. |
|  | *Speaking in public* | 0.10 | Do you feel comfortable speaking up in public to help decide on infrastructure (like small wells, roads) to be built, to ensure proper payment of wages for public work or other similar programs, or to protest the misbehavior of authorities or elected officials? | Achievement in any. Inadequate if not comfortable speaking in public. | Adequacy is calculated as the weighted average of the levels of comfort associated with all three public speaking contexts. | Adequacy is calculated as the weighted average of the levels of comfort associated with the context in which she participates. |
| **Time** | *Workload* | 0.10 | Worked more than 10.5 hours in the previous 24 hours. | Inadequate if individual works more than 10.5 hours per day. | Inadequate if individual works more than 10.5 hours per day. | Inadequate if individual works more than 10.5 hours per day. |
|  | *Leisure* | 0.10 | How would you rate your satisfaction with your time available for leisure activities such as visiting neighbors, watching TV, listening to the radio, seeing movies, or doing sports? | Inadequate if not  satisfied (<5); 5 being neither satisfied not dissatisfied. | Adequacy is calculated as the weighted average of the levels of satisfaction. | Inadequate if not  satisfied (<5); 5 being neither satisfied not dissatisfied. |

Source: Alkire et al. [1] & authors
