# Supplementary material for: Women’s empowerment in agriculture and productivity change: The case of Bangladesh rice farms
Source: PLoS One. 2021 Aug 4;16(8):e0255589. doi: 10.1371/journal.pone.0255589 (PMC8336850; doi:10.1371/journal.pone.0255589)
Supplement: S2 File — (DOCX) [file pone.0255589.s002.docx]

**Online supplement S2**

This section discusses the control variable results of table 6 of the main text. Sex of the primary decision maker is negatively associated with technical change indicating female-headed households make, on average, less technological investments in their farm operations than male headed households. This could be due to the fact that women have relatively poor access to productive resources, inputs and services [2,3]. Education positively affects both the technical change and scale change index. A reason could be that education reduces barriers to adoption and implementation of new agricultural technologies that can not only help farm households to shift their production frontier upward but also bring them closer to the optimal scale. Results also indicate that the dependency ratio negatively affects productivity change and efficiency change. One possible explanation is that a higher dependency ratio replaces unpaid family labor with paid hired labor, which essentially lowers farms' economic performance as overall production cost rises due to reduced economics of scale. Household size is negatively associated with efficiency change and scale change implying that with the increase in the size of the household, a household’s ability to use inputs more efficiently and expand the scale of the farm operations diminishes. It might be the case that households with more people spend more resources for their increased consumption needs and save less, limiting their ability to increase productivity at intensive (yield growth through efficient input mix) and extensive (production growth through land expansion) margins. On the other hand, an increase in the share of household income from non-agricultural sources induces a positive shift in efficiency change. This may be due to the fact that an increase in off-farm income increases a household’s liquidity or access to credit, facilitating farm investment, including in better inputs (e.g., purchasing quality seeds, fertilizer), which helps farms to use better technology. The coefficient on irrigation (rainfed) does not have a statistically significant effect. This result differs from expectations based on at least some past studies [4,5], although comparisons are complicated because some past studies focus on a different measure of productivity such as yield. Moving to the tenancy variables, we found a statistically significant, positive effect on productivity change and technical change index. Results indicate that overall productivity increases when the plot(s) is taken-in through a cash lease or crop-sharing arrangement. The positive relationship between tenancy and technical change implies that technological improvement differs depending on the operational status of the arable land. On average, technological improvement is higher in the lands operated under cash lease or crop-sharing arrangement compared to the lands operated under different tenure systems such as owner-operated, group leasing, or joint operation. Both weather variables have a statistically significant impact on productivity indices. The rainfall change variable negatively affects the productivity change and technical change index, but the effect is positive for scale efficiency change. The temperature change variable has a positive effect on productivity, efficiency, and technical change index. Among the locational dummies, households located in northwestern, northeastern, and southwestern districts were found to exhibit technological progress and regress, respectively, compared to households located in the southeastern districts of the country. Households situated in the northeastern districts were also found to experience productivity growth and efficiency gains compared to those in the southeastern districts of the country.
