# Supplementary material for: Women’s empowerment in agriculture and productivity change: The case of Bangladesh rice farms
Source: PLoS One. 2021 Aug 4;16(8):e0255589. doi: 10.1371/journal.pone.0255589 (PMC8336850; doi:10.1371/journal.pone.0255589)
Supplement: S3 File — (DOCX) [file pone.0255589.s003.docx]

**Online supplement S3**

Table 1. Results of the OLS bootstrap regression of the determinants of farm productivity change and its components (Empowerment gap)

|  | Model 1: Empowerment gap based on Alkire et al. [6] | | | | Model 2: Alternative empowerment gap based on approach 1 | | | | Model 3: Alternative empowerment gap based on approach 2 | | | |
| --- | --- | --- | --- | --- | --- | --- | --- | --- | --- | --- | --- | --- |
| Variables | Productivity change | Efficiency change | Technical change | Scale efficiency change | Productivity change | Efficiency change | Technical change | Scale efficiency change | Productivity change | Efficiency change | Technical change | Scale efficiency change |
| Empowerment gap | -0.128** | -0.105 | -0.027 | 0.002 | -0.182* | -0.224 | -0.016 | -0.007 | -0.117 | -0.123 | -0.013 | -0.001 |
|  | (0.063) | (0.094) | (0.018) | (0.030) | (0.109) | (0.157) | (0.028) | (0.054) | (0.077) | (0.112) | (0.021) | (0.033) |
| Sex | -0.097 | -0.085 | -0.034* | 0.018 | -0.102 | -0.093 | -0.034* | 0.018 | -0.102 | -0.091 | -0.034* | 0.018 |
|  | (0.070) | (0.107) | (0.020) | (0.025) | (0.071) | (0.110) | (0.019) | (0.026) | (0.069) | (0.107) | (0.020) | (0.026) |
| Age | 0.001 | 0.001 | 0.000 | 0.000 | 0.001 | 0.001 | 0.000 | 0.000 | 0.001 | 0.001 | 0.000 | 0.000 |
|  | (0.001) | (0.001) | (0.000) | (0.000) | (0.001) | (0.001) | (0.000) | (0.000) | (0.001) | (0.001) | (0.000) | (0.000) |
| Education | 0.002 | -0.004 | 0.002* | 0.004* | 0.002 | -0.003 | 0.002* | 0.004 | 0.002 | -0.004 | 0.002* | 0.004* |
|  | (0.003) | (0.005) | (0.001) | (0.002) | (0.003) | (0.005) | (0.001) | (0.003) | (0.003) | (0.005) | (0.001) | (0.002) |
| Dependency ratio | -0.049** | -0.056** | -0.008 | -0.006 | -0.049** | -0.057** | -0.008 | -0.006 | -0.049** | -0.056** | -0.008 | -0.006 |
|  | (0.019) | (0.028) | (0.005) | (0.011) | (0.019) | (0.028) | (0.005) | (0.012) | (0.019) | (0.028) | (0.005) | (0.011) |
| Household size | -0.014 | -0.020* | 0.001 | -0.005** | -0.014 | -0.020* | 0.001 | -0.005** | -0.014 | -0.020* | 0.001 | -0.005** |
|  | (0.009) | (0.011) | (0.002) | (0.002) | (0.009) | (0.012) | (0.003) | (0.002) | (0.009) | (0.011) | (0.003) | (0.002) |
| Extension visit | 0.013 | -0.004 | 0.007 | 0.004 | 0.013 | -0.004 | 0.007 | 0.004 | 0.013 | -0.004 | 0.007 | 0.004 |
|  | (0.021) | (0.013) | (0.009) | (0.004) | (0.021) | (0.013) | (0.010) | (0.004) | (0.021) | (0.013) | (0.010) | (0.004) |
| Income share from non ag. enterprise | 0.052 | 0.113** | -0.010 | -0.010 | 0.054 | 0.115** | -0.010 | -0.010 | 0.053 | 0.114** | -0.010 | -0.010 |
|  | (0.037) | (0.055) | (0.009) | (0.017) | (0.036) | (0.055) | (0.009) | (0.017) | (0.037) | (0.055) | (0.009) | (0.018) |
| Rainfed | 0.023 | 0.028 | 0.002 | -0.004 | 0.025 | 0.031 | 0.002 | -0.004 | 0.023 | 0.028 | 0.001 | -0.004 |
|  | (0.025) | (0.035) | (0.006) | (0.012) | (0.024) | (0.035) | (0.006) | (0.011) | (0.025) | (0.035) | (0.006) | (0.011) |
| Tenancy | 0.061** | 0.059 | 0.012** | 0.011 | 0.060** | 0.059 | 0.012** | 0.011 | 0.060** | 0.059 | 0.012** | 0.011 |
|  | (0.026) | (0.037) | (0.005) | (0.017) | (0.026) | (0.037) | (0.005) | (0.018) | (0.026) | (0.037) | (0.005) | (0.017) |
| Rainfall | -0.000* | -0.000 | -0.000*** | 0.000* | -0.000** | -0.000 | -0.000*** | 0.000* | -0.000** | -0.000 | -0.000*** | 0.000* |
|  | (0.000) | (0.000) | (0.000) | (0.000) | (0.000) | (0.000) | (0.000) | (0.000) | (0.000) | (0.000) | (0.000) | (0.000) |
| Temperature | 0.249*** | 0.222 | 0.070*** | -0.007 | 0.251*** | 0.233* | 0.067*** | -0.005 | 0.246*** | 0.224* | 0.067*** | -0.006 |
|  | (0.091) | (0.135) | (0.024) | (0.026) | (0.091) | (0.135) | (0.023) | (0.025) | (0.090) | (0.134) | (0.024) | (0.026) |
| Northwest | 0.033 | -0.019 | 0.024*** | 0.035 | 0.037 | -0.014 | 0.025*** | 0.035 | 0.032 | -0.019 | 0.024*** | 0.035 |
|  | (0.034) | (0.047) | (0.008) | (0.024) | (0.034) | (0.048) | (0.008) | (0.025) | (0.034) | (0.048) | (0.008) | (0.024) |
| Southwest | -0.050 | -0.004 | -0.029*** | 0.001 | -0.048 | -0.000 | -0.029*** | 0.001 | -0.053 | -0.006 | -0.030*** | 0.001 |
|  | (0.038) | (0.054) | (0.010) | (0.010) | (0.038) | (0.055) | (0.011) | (0.010) | (0.038) | (0.053) | (0.011) | (0.010) |
| Northeast | 0.165*** | 0.150* | 0.052*** | -0.024 | 0.166*** | 0.153* | 0.052*** | -0.023 | 0.170*** | 0.156* | 0.053*** | -0.024 |
|  | (0.059) | (0.087) | (0.015) | (0.022) | (0.058) | (0.088) | (0.014) | (0.022) | (0.058) | (0.089) | (0.015) | (0.022) |
| Constant | 0.635*** | 1.099*** | 0.583*** | 1.051*** | 0.630*** | 1.095*** | 0.582*** | 1.051*** | 0.634*** | 1.098*** | 0.583*** | 1.051*** |
|  | (0.083) | (0.119) | (0.020) | (0.036) | (0.082) | (0.119) | (0.021) | (0.035) | (0.084) | (0.120) | (0.021) | (0.036) |

Note: Standard errors in parentheses; *, **, and *** indicate significance based on 90%, 95% and 99% bootstrap confidence level, respectively

Table 2. Results of the OLS bootstrap regression of the determinants of farm productivity change and its components (WEAI domains)

|  | Model 1: Empowerment domain score based on Alkire et al. [6] | | | | Model 2: Alternative empowerment domain score based on approach 1 | | | | Model 3: Alternative empowerment domain score based on approach 2 | | | | |
| --- | --- | --- | --- | --- | --- | --- | --- | --- | --- | --- | --- | --- | --- |
| Variables | Productivity change | Efficiency change | Technical change | Scale efficiency change | Productivity change | Efficiency change | Technical change | Scale efficiency change | Productivity change | Efficiency change | Technical change | Scale efficiency change |  |
| Empowerment score: Production | 0.483*** | 0.458* | 0.115*** | 0.038 | 0.798*** | 0.885*** | 0.121** | 0.147 | 0.762*** | 0.838*** | 0.120** | 0.132 |  |
|  | (0.168) | (0.241) | (0.041) | (0.090) | (0.233) | (0.329) | (0.054) | (0.179) | (0.217) | (0.314) | (0.049) | (0.174) |  |
| Empowerment score: Resource | 0.064 | 0.091 | 0.041 | -0.067 | 0.843 | 0.919 | -0.080 | 0.677 | 0.412** | 0.500* | 0.047 | 0.014 |  |
|  | (0.179) | (0.265) | (0.042) | (0.058) | (0.593) | (0.837) | (0.142) | (0.542) | (0.199) | (0.303) | (0.049) | (0.084) |  |
| Empowerment score: Income | -0.010 | -0.075 | -0.021 | 0.026 | -0.091 | -0.255 | 0.124 | -0.311 | -0.101 | -0.181 | 0.014 | -0.132 |  |
|  | (0.140) | (0.211) | (0.034) | (0.044) | (0.413) | (0.583) | (0.083) | (0.247) | (0.239) | (0.336) | (0.054) | (0.124) |  |
| Empowerment score: Leadership | 0.096 | 0.224 | 0.033 | -0.196* | 0.314 | 0.576 | 0.047 | -0.472 | -0.087 | 0.092 | -0.024 | -0.217 |  |
|  | (0.205) | (0.275) | (0.053) | (0.103) | (0.574) | (0.806) | (0.118) | (0.348) | (0.249) | (0.330) | (0.064) | (0.133) |  |
| Empowerment score: Time | 0.196 | 0.174 | 0.016 | 0.083 | 0.253 | 0.293 | 0.008 | 0.112 | 0.187 | 0.167 | 0.017 | 0.077 |  |
|  | (0.172) | (0.258) | (0.042) | (0.078) | (0.205) | (0.298) | (0.049) | (0.119) | (0.171) | (0.255) | (0.042) | (0.080) |  |
| Sex | -0.113 | -0.102 | -0.039** | 0.018 | -0.123* | -0.110 | -0.038** | 0.012 | -0.125* | -0.117 | -0.039* | 0.020 |  |
|  | (0.070) | (0.110) | (0.020) | (0.026) | (0.073) | (0.113) | (0.019) | (0.027) | (0.072) | (0.112) | (0.020) | (0.026) |  |
| Age | 0.001 | 0.001 | 0.000 | -0.000 | 0.001 | 0.001 | 0.000 | 0.000 | 0.001 | 0.001 | 0.000 | 0.000 |  |
|  | (0.001) | (0.001) | (0.000) | (0.000) | (0.001) | (0.001) | (0.000) | (0.000) | (0.001) | (0.001) | (0.000) | (0.000) |  |
| Education | 0.001 | -0.004 | 0.001* | 0.004* | 0.001 | -0.005 | 0.002* | 0.004* | 0.001 | -0.005 | 0.001* | 0.004* |  |
|  | (0.003) | (0.005) | (0.001) | (0.002) | (0.003) | (0.005) | (0.001) | (0.002) | (0.003) | (0.005) | (0.001) | (0.002) |  |
| Dependency ratio | -0.046** | -0.053* | -0.007 | -0.007 | -0.045** | -0.051* | -0.008* | -0.005 | -0.047** | -0.053* | -0.007 | -0.006 |  |
|  | (0.019) | (0.028) | (0.005) | (0.011) | (0.019) | (0.029) | (0.005) | (0.011) | (0.019) | (0.028) | (0.005) | (0.011) |  |
| Household Size | -0.014 | -0.021* | 0.001 | -0.005* | -0.014 | -0.020* | 0.001 | -0.005* | -0.013 | -0.019 | 0.001 | -0.005* |  |
|  | (0.009) | (0.012) | (0.002) | (0.002) | (0.009) | (0.012) | (0.002) | (0.003) | (0.009) | (0.012) | (0.003) | (0.002) |  |
| Extension visit | 0.013 | -0.005 | 0.007 | 0.004 | 0.012 | -0.006 | 0.007 | 0.004 | 0.012 | -0.005 | 0.007 | 0.004 |  |
|  | (0.021) | (0.013) | (0.010) | (0.004) | (0.021) | (0.013) | (0.010) | (0.004) | (0.021) | (0.013) | (0.010) | (0.004) |  |
| Income share from non ag. enterprise | 0.054 | 0.114** | -0.010 | -0.008 | 0.054 | 0.115** | -0.010 | -0.008 | 0.058 | 0.118** | -0.009 | -0.007 |  |
|  | (0.037) | (0.055) | (0.009) | (0.017) | (0.037) | (0.055) | (0.009) | (0.017) | (0.037) | (0.055) | (0.009) | (0.017) |  |
| Rainfed | -0.018 | -0.011 | -0.004 | -0.009 | -0.018 | -0.010 | -0.003 | -0.011 | -0.019 | -0.012 | -0.004 | -0.010 |  |
|  | (0.024) | (0.034) | (0.006) | (0.011) | (0.024) | (0.034) | (0.006) | (0.012) | (0.024) | (0.034) | (0.006) | (0.012) |  |
| Tenancy | 0.049* | 0.030 | 0.015*** | 0.016 | 0.049* | 0.029 | 0.015*** | 0.017 | 0.050** | 0.031 | 0.015*** | 0.015 |  |
|  | (0.025) | (0.036) | (0.006) | (0.018) | (0.025) | (0.036) | (0.005) | (0.019) | (0.025) | (0.036) | (0.006) | (0.018) |  |
| Rainfall | -0.000 | -0.000 | -0.000*** | 0.000* | -0.000* | -0.000 | -0.000*** | 0.000 | -0.000* | -0.000 | -0.000*** | 0.000* |  |
|  | (0.000) | (0.000) | (0.000) | (0.000) | (0.000) | (0.000) | (0.000) | (0.000) | (0.000) | (0.000) | (0.000) | (0.000) |  |
| Temperature | 0.236*** | 0.214 | 0.070*** | -0.015 | 0.246*** | 0.227* | 0.064*** | 0.004 | 0.241*** | 0.225 | 0.067*** | -0.013 |  |
|  | (0.091) | (0.136) | (0.024) | (0.027) | (0.091) | (0.137) | (0.023) | (0.025) | (0.090) | (0.137) | (0.024) | (0.027) |  |
| Northwest | 0.040 | -0.015 | 0.026*** | 0.039 | 0.044 | -0.008 | 0.028*** | 0.034 | 0.049 | -0.006 | 0.027*** | 0.040 |  |
|  | (0.034) | (0.047) | (0.008) | (0.027) | (0.034) | (0.048) | (0.008) | (0.024) | (0.035) | (0.048) | (0.008) | (0.029) |  |
| Southwest | -0.035 | 0.008 | -0.027** | 0.007 | -0.032 | 0.013 | -0.024** | -0.002 | -0.031 | 0.012 | -0.026** | 0.007 |  |
|  | (0.038) | (0.054) | (0.011) | (0.011) | (0.038) | (0.056) | (0.010) | (0.010) | (0.039) | (0.054) | (0.011) | (0.012) |  |
| Northeast | 0.156*** | 0.142* | 0.054*** | -0.027 | 0.174*** | 0.162* | 0.049*** | -0.005 | 0.174*** | 0.166* | 0.054*** | -0.023 |  |
|  | (0.057) | (0.086) | (0.015) | (0.022) | (0.058) | (0.087) | (0.015) | (0.022) | (0.057) | (0.087) | (0.015) | (0.022) |  |
| Constant | 0.554*** | 1.028*** | 0.563*** | 1.056*** | 0.514*** | 0.971*** | 0.571*** | 1.014*** | 0.490*** | 0.940*** | 0.560*** | 1.050*** |  |
|  | (0.092) | (0.136) | (0.025) | (0.036) | (0.089) | (0.130) | (0.023) | (0.037) | (0.092) | (0.135) | (0.024) | (0.035) |  |

Note: Standard errors in parentheses; *, **, and *** indicate significance based on 90%, 95% and 99% bootstrap confidence level, respectively

Table 3. Results of the OLS bootstrap regression of the determinants of farm productivity change and its components (production domain indicators)

|  | Model 1: Production domain indicator based on Alkire et al. [6] | | | | Model 2: Alternative production domain indicator based on approach 1 | | | | Model 3: Alternative production domain indicator based on approach 2 | | | | |
| --- | --- | --- | --- | --- | --- | --- | --- | --- | --- | --- | --- | --- | --- |
| Variables | Productivity change | Efficiency change | Technical change | Scale efficiency change | Productivity change | Efficiency change | Technical change | Scale efficiency change | Productivity change | Efficiency change | Technical change | Scale efficiency change |  |
| Input in productive decisions | 0.045 | -0.220 | 0.087 | -0.017 | 0.564 | -0.102 | 0.390*** | 0.040 | 0.175 | -0.058 | 0.071 | 0.093 |  |
|  | (0.237) | (0.351) | (0.064) | (0.087) | (0.712) | (0.993) | (0.144) | (0.308) | (0.484) | (0.713) | (0.113) | (0.269) |  |
| Autonomy in production | 0.944*** | 1.121*** | 0.133** | 0.094 | 0.896*** | 1.101*** | 0.107* | 0.088 | 0.945*** | 1.093*** | 0.143** | 0.089 |  |
|  | (0.239) | (0.338) | (0.056) | (0.130) | (0.240) | (0.345) | (0.057) | (0.120) | (0.230) | (0.333) | (0.056) | (0.129) |  |
| Sex | -0.099 | -0.082 | -0.036* | 0.017 | -0.103 | -0.085 | -0.038** | 0.016 | -0.101 | -0.085 | -0.036* | 0.015 |  |
|  | (0.071) | (0.112) | (0.019) | (0.025) | (0.073) | (0.113) | (0.019) | (0.026) | (0.070) | (0.112) | (0.019) | (0.027) |  |
| Age | 0.001 | 0.001 | 0.000 | 0.000 | 0.001 | 0.001 | 0.000 | 0.000 | 0.001 | 0.001 | 0.000 | 0.000 |  |
|  | (0.001) | (0.001) | (0.000) | (0.000) | (0.001) | (0.001) | (0.000) | (0.000) | (0.001) | (0.001) | (0.000) | (0.000) |  |
| Education | 0.001 | -0.004 | 0.002* | 0.004* | 0.001 | -0.005 | 0.002* | 0.004* | 0.001 | -0.005 | 0.002* | 0.004* |  |
|  | (0.003) | (0.005) | (0.001) | (0.002) | (0.003) | (0.005) | (0.001) | (0.002) | (0.003) | (0.005) | (0.001) | (0.002) |  |
| Dependency ratio | -0.046** | -0.052* | -0.007 | -0.006 | -0.047** | -0.053* | -0.008* | -0.006 | -0.046** | -0.053* | -0.007 | -0.006 |  |
|  | (0.019) | (0.028) | (0.005) | (0.011) | (0.019) | (0.028) | (0.005) | (0.011) | (0.019) | (0.028) | (0.005) | (0.011) |  |
| Household Size | -0.016* | -0.022* | 0.001 | -0.006** | -0.015* | -0.022* | 0.001 | -0.005** | -0.016* | -0.022* | 0.001 | -0.005** |  |
|  | (0.009) | (0.012) | (0.002) | (0.003) | (0.009) | (0.012) | (0.002) | (0.003) | (0.009) | (0.011) | (0.003) | (0.003) |  |
| Extension visit | 0.012 | -0.006 | 0.007 | 0.004 | 0.012 | -0.006 | 0.007 | 0.004 | 0.012 | -0.006 | 0.007 | 0.004 |  |
|  | (0.021) | (0.012) | (0.010) | (0.004) | (0.021) | (0.013) | (0.010) | (0.004) | (0.021) | (0.013) | (0.010) | (0.004) |  |
| Income share from non ag. enterprise | 0.061* | 0.126** | -0.009 | -0.010 | 0.060 | 0.125** | -0.010 | -0.010 | 0.062* | 0.125** | -0.009 | -0.010 |  |
|  | (0.037) | (0.056) | (0.009) | (0.017) | (0.037) | (0.055) | (0.009) | (0.018) | (0.037) | (0.054) | (0.009) | (0.017) |  |
| Rainfed | -0.018 | -0.010 | -0.004 | -0.010 | -0.017 | -0.010 | -0.004 | -0.010 | -0.018 | -0.009 | -0.005 | -0.010 |  |
|  | (0.024) | (0.034) | (0.006) | (0.012) | (0.024) | (0.034) | (0.006) | (0.011) | (0.024) | (0.035) | (0.006) | (0.011) |  |
| Tenancy | 0.051** | 0.032 | 0.015*** | 0.016 | 0.051** | 0.032 | 0.015*** | 0.016 | 0.051** | 0.032 | 0.015*** | 0.016 |  |
|  | (0.025) | (0.036) | (0.006) | (0.018) | (0.025) | (0.036) | (0.005) | (0.018) | (0.025) | (0.035) | (0.006) | (0.018) |  |
| Rainfall | -0.000* | -0.000 | -0.000*** | 0.000* | -0.000 | -0.000 | -0.000*** | 0.000* | -0.000* | -0.000 | -0.000*** | 0.000* |  |
|  | (0.000) | (0.000) | (0.000) | (0.000) | (0.000) | (0.000) | (0.000) | (0.000) | (0.000) | (0.000) | (0.000) | (0.000) |  |
| Temperature | 0.204** | 0.167 | 0.066*** | -0.008 | 0.205** | 0.172 | 0.066*** | -0.008 | 0.204** | 0.172 | 0.065*** | -0.007 |  |
|  | (0.090) | (0.133) | (0.023) | (0.026) | (0.089) | (0.134) | (0.023) | (0.027) | (0.089) | (0.134) | (0.023) | (0.026) |  |
| Northwest | 0.037 | -0.015 | 0.026*** | 0.036 | 0.040 | -0.015 | 0.028*** | 0.036 | 0.038 | -0.015 | 0.026*** | 0.037 |  |
|  | (0.034) | (0.047) | (0.008) | (0.025) | (0.035) | (0.048) | (0.008) | (0.026) | (0.035) | (0.048) | (0.008) | (0.026) |  |
| Southwest | -0.042 | 0.001 | -0.027*** | 0.002 | -0.036 | 0.002 | -0.024** | 0.002 | -0.041 | 0.003 | -0.027** | 0.003 |  |
|  | (0.037) | (0.055) | (0.010) | (0.010) | (0.038) | (0.054) | (0.010) | (0.011) | (0.037) | (0.054) | (0.011) | (0.011) |  |
| Northeast | 0.158*** | 0.144* | 0.052*** | -0.023 | 0.155*** | 0.143 | 0.050*** | -0.024 | 0.158*** | 0.142 | 0.053*** | -0.024 |  |
|  | (0.057) | (0.085) | (0.015) | (0.021) | (0.058) | (0.089) | (0.015) | (0.022) | (0.057) | (0.087) | (0.015) | (0.021) |  |
| Constant | 0.612*** | 1.092*** | 0.572*** | 1.049*** | 0.600*** | 1.082*** | 0.567*** | 1.047*** | 0.603*** | 1.083*** | 0.572*** | 1.042*** |  |
|  | (0.083) | (0.120) | (0.021) | (0.033) | (0.083) | (0.123) | (0.021) | (0.033) | (0.086) | (0.129) | (0.022) | (0.029) |  |

Note: Standard errors in parentheses; *, **, and *** indicate significance based on 90%, 95% and 99% bootstrap confidence level, respectively

**References**

1. Alkire S, Malapit H, Meinzen-Dick R, Peterman A, Quisumbing A, Seymour G, et al. Instructional Guide on the Women’s Empowerment in Agriculture Index. Washington, DC: International Food Policy Research Institute (IFPRI); 2013 p. 82.

2. Food and Agriculture Organization. Women in agriculture: closing the gender gap for development. Rome, Italy; 2011.

3. Quisumbing AR. Male-female differences in agricultural productivity: Methodological issues and empirical evidence. World Dev. 1996;24: 1579–1595. doi:10.1016/0305-750X(96)00059-9

4. M. Rahman W, L. Parvin. Impact of Irrigation on Food Security in Bangladesh for the Past Three Decades. J Water Resour Prot. 2009;2009. doi:10.4236/jwarp.2009.13027

5. Palmer-Jones RW. Sustaining Serendipity? Groundwater Irrigation, Growth of Agricultural Production, and Poverty in Bangladesh. Econ Polit Wkly. 1992;27: A128–A140.

6. Alkire S, Meinzen-Dick R, Peterman A, Quisumbing A, Seymour G, Vaz A. The Women’s Empowerment in Agriculture Index. World Dev. 2013;52: 71–91. doi:10.1016/j.worlddev.2013.06.007
